# Increased fibrotic signaling in a murine model for intra-arterial contrast-induced acute kidney injury

**DOI:** 10.1152/ajprenal.00004.2020

**Published:** 2020-03-23

**Authors:** Amit Sharma, Sreenivasulu Kilari, Chuanqi Cai, Michael L. Simeon, Sanjay Misra

**Affiliations:** ^1^Vascular and Interventional Radiology Translational Laboratory, Department of Radiology, Mayo Clinic, Rochester, Minnesota; ^2^Department of Vascular Surgery, Union Hospital, Tongji Medical College, Huazhong University of Science and Technology, Wuhan, China; ^3^Department of Radiology, Mayo Clinic, Rochester, Minnesota; ^4^Department of Biochemistry and Molecular Biology, Mayo Clinic, Rochester, Minnesota

**Keywords:** animal models, contrast, kidney, creatinine, kidney injury molecule-1, postcontrast acute kidney injury, transforming growth factor-β_1_/SMAD3 signaling

## Abstract

Contrast-induced acute kidney injury (CI-AKI) is a vexing problem, and more than 70 million patients undergo studies using iodinated contrast. The molecular mechanisms responsible for CI-AKI are poorly understood. The goal of the present article was to determine the role of transforming growth factor-β1 (TGF-β1)/mothers against decapentaplegic homolog (SMAD)3 and associated collagen expression in a murine model of intra-arterial CI-AKI. The murine model of CI-AKI after intra-arterial contrast agent administration was created by first performing a partial nephrectomy to induce chronic kidney disease. Twenty-eight days later, 100 μL of contrast agent [iodixanol (320 mg/mL)] or saline were administered via the carotid artery. Two days after contrast administration, compared with saline, average serum creatinine was significantly elevated (*P* < 0.05). In the cortex, there was a significant increase in phosphorylated SMAD3 and gene expression of TGF-β1, TGF-β receptor type I, and TGF-β receptor type II at *day 2* in the contrast group compared with the saline group. Average gene expressions of connective tissue growth factor, matrix metalloproteinase-2 and −9, and collagen type I-α and type IV-α were significantly increased at 2 days after contrast administration (all *P* < 0.05). Moreover, there was a decrease in Ki-67 staining in the cortex, with an increase in terminal deoxynucleotidyl transferase dUTP nick-end labeling in the cortex and medulla after contrast administration (*P* < 0.05). In the murine intra-arterial CI-AKI model, there was increased hypoxia and TGF-β1/SMAD3 pathway activation and collagen expression, resulting in renal fibrosis. Together, these results suggest that the TGF-β1/SMAD3 pathway could be a potential target in alleviating tissue fibrosis in CI-AKI.

## INTRODUCTION

Contrast-induced acute kidney injury (CI-AKI), often referred as contrast-induced nephropathy, is a clinical problem for physicians using iodinated contrast agent (25, 36). The Acute Kidney Injury Network defines CI-AKI as an increase in serum creatinine (SCr) of ≥0.3 mg/dL with oliguria (22). The occurrence of CI-AKI is estimated to be up to 15% of the general population receiving intravascular iodinated contrast agents (20). Multiple studies have demonstrated that the CI-AKI risk is minimal in patients with intravenous contrast agent administration (10, 36) compared with intra-arterial contrast administration, which is as high as 10–30% (36, 37). However, a recent clinical study by McDonald et al. (21) concluded that the CI-AKI risk is independent of the route of contrast administration but that the CI-AKI risk varies with other baseline clinical factors. In support of this notion, it has been proposed that the CI-AKI risk increases to 50% in patients with multiple comorbidities, including chronic kidney disease (CKD), diabetes, heart failure, anemia, and hemodynamic instability (30).

Although histological analysis of the kidney with CI-AKI demonstrates evidence of tissue fibrosis, the mechanisms responsible for the development of CI-AKI are poorly understood. It is hypothesized that contrast causes vasoconstriction and hypoxic injury (8, 9), resulting in tubular fibrosis (1, 14). A recent study from our laboratory demonstrated that increased transforming growth factor-β1 (TGF-β1)/SMAD3 signaling after intravenous contrast administration to mice with established CKD (11). The purpose of the present study was to investigate the pathophysiology of CI-AKI after the intra-arterial contrast agent administration in an established CKD mouse model. Kidney function was assessed by measuring SCr, blood urea nitrogen (BUN), and urinary kidney injury molecule (KIM)-1 at 2 and 7 days after contrast administration. Moreover, histological examination and gene expression experiments were performed to assess tissue fibrosis, hypoxia, phosphorylated (p)SMAD3 signaling, proliferation, and apoptosis in the renal cortex and medulla.

## MATERIALS AND METHODS

All chemicals and assay kits were purchased from Sigma-Aldrich unless otherwise mentioned.

### 

#### Animal study design.

**Fig. 1.**
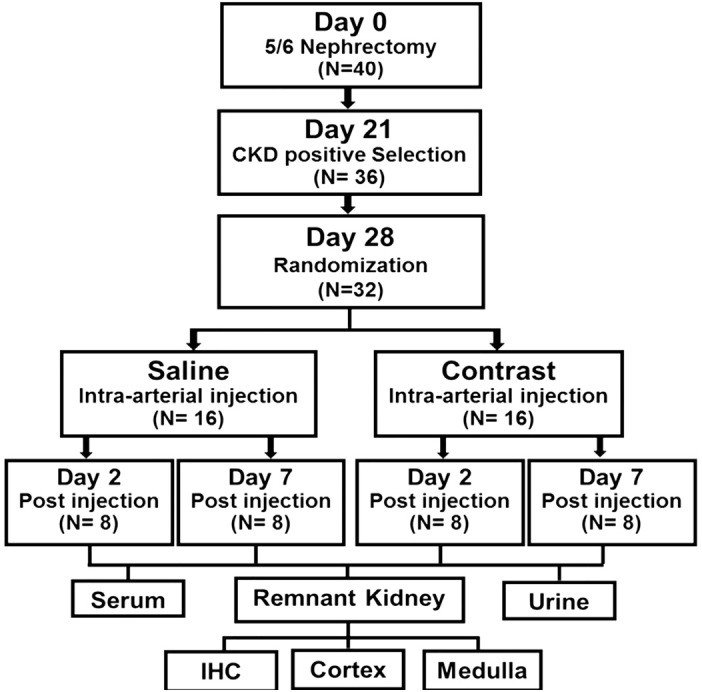
Schematic representation of the study design. CKD, chronic kidney disease; IHC, immunohistochemistry.

The animal study plan is shown in Fig. 1. The Mayo Clinic Institutional Animal Care and Use Committee approval was obtained before any animal procedures were performed. All mouse procedures were conducted according to the Public Health Service Policy on Humane Care and Use of Laboratory Animals. Male C57BL/6J mice weighing 19–25 g were obtained from Jackson Laboratories (Bar Harbor, ME) and housed at 22°C, 41% relative humidity, and 12:12-h light-dark cycles. CKD was induced by performing a 5/6 renal nephrectomy, as previously described (13).

#### Contrast administration.

Twenty-eight days after nephrectomy, animals were randomized to receive either 100 μL of saline or radiocontrast [iodixanol (320 mg/mL), GE Healthcare, Waukesha, WI) administered through the right carotid artery. Mice were anesthetized with 2–4% isoflurane, and the right common carotid artery was exposed with a small incision, as previously described (34). A polyethylene capillary catheter (catalog no. 427410, Intramedic, VWR, Radnor, PA) was inserted into the carotid artery, and 100 µL of radiocontrast (CI group) or saline (saline group) was injected at a flow rate of 10 µL/min using a syringe pump. The vessel was ligated using 10-0 monofilament nylon sutures (AK-2120, Surgical Specialties, Tijuana, Mexico), and the incision was closed using 6-0 suture (Polyglycan 910, Ethicon, Miami, FL).

At 2 or 7 days after contrast or saline administration, mice were euthanized, and blood and the remnant kidney were collected. In addition, blood and urine were collected weekly using the submandibular vein puncture and spot collection method, respectively. Serum was separated from the blood. Urine and serum samples were stored at −80°C until analyzed. The remnant kidney was dissected in a sagittal plane into two halves. One half of the kidney was preserved in 10% buffered formalin for histology. The other half of the kidney was separated into the cortex and medulla and stored at −80°C for gene expression and protein analyses.

#### Biochemical analysis.

BUN (BioAssay Systems, Hayward, CA), and creatinine (Cayman Chemical, Ann Arbor, MI) levels were measured using commercial kits. Urinary KIM-1 was measured using an ELISA kit (catalog. no. MKM100, R&D Systems, Minneapolis, MN) following the manufacturer’s protocol.

#### Histopathology, immunohistochemistry, and image analysis.

Paraffin-embedded tissue blocks were cut into 5-μm-thick sections and immunostained. The antibodies and dilutions used are shown in Table 1. Picrosirus red (PSR) staining was performed to assess interstitial fibrosis. All images were captured at ×200 magnification using Carl Zeiss Imager M2 (Thornwood, NY) equipped with a motorized stage and Axiocam 503 camera. The analysis for the histological images was performed as follows. The histological evaluation was performed by a person blinded to the treatment group. The entire tissue section was selected as the region of interest to measure the total tissue area and area of tissue that stained either brown (immunohistochemistry) or red (PSR) was selected. The percent index of brown- or red-positive stain in the total tissue area was calculated (4a).

**Table 1.** Antibodies used for histology

Antibody/Assay Kit	Supplier	Catalog Number	Dilution
TGFβR1	Abcam	ab31013	1:500
E-cadherin	Abcam	ab231303	1:500
HIF-1α	Novus Biologicals	NB100-105	1:1,000
FSP-1	EMD Millipore	07-2274	1:500
Ki-67	EMD Millipore	ab9260	1:800
TUNEL	ThermoFisher Scientific	4810-30-K	As instructed
pSMAD3	Cell Signaling Technology	9520	1:1,000

TGFβR1, transforming growth factor-β receptor type I; HIF-1α, hypoxia-inducible factor-1α; FSP-1, fibroblast-specific protein-1; pSMAD3, phosphorylated SMAD3.

#### Real-time PCR.

Quantitative real-time PCR analysis was performed to assess the changes in gene expression of profibrotic genes, as previously described (14). mRNA was isolated from the renal cortex and medulla using the RNeasy mini kit (Qiagen, Hilden, Germany), and cDNA was prepared using the iScript select cDNA synthesis kit (Bio-Rad Laboratories, Hercules, CA). Quantitative real-time PCR was performed using a C1000 Thermal cycler equipped with a CFX96 optical system using iTaq universal SYBR Green Master Mix (Bio-Rad). Each sample was run in triplicate, and threshold cycle (C_t_) values were normalized to 18S. The fold change in gene expression was calculated following the $2^{-(\Delta \Delta {\text{C}}_{\text{t}})}$ method. The primers used for quantitative real-time PCRs are shown in Table 2.

**Table 2.** Primers used for quantitative RT-PCR gene expression analysis

Gene	Forward	Reverse
18S	5′-GTTCCACATAAACGATGCC-3′	5′-TGGTGGTGCCCTTCCGTCAAT-3′
HIF-1α	5′-CCAATTCCTCATCCGTCAATTA-3′	5′-GGCTCATAACCCATCAACTCA-3′
TGF-β1	5′-CGAAGCGGACTACTATGCTAAA-3′	5′-TCCCGAATGTCTGACGTATTG-3′
TGFβR1	5′-TTGCTCCAAACCACAGAGTAG-3′	5′-ACACTAAGCCCATTGCATAGT-3′
TGFβR2	5′-GTGCCTGTAACATGGAAGAGT-3′	5′-ACACCCGTCACTTGGATAATG-3′
CTGF	5′-GATGCACTTTTTGCCCTTCTTAATG-3′	5′-CACAGAGTGGAGCGCCTGTTC-3′
Col-Ia	5′-CATAAAGGGTCATCGTGGCT-3′	5′-TTGAGTCCGTCTTTGCCAG-3′
Col-IVa	5′-CACCCATCTCTGGGGACAAC-3′	5′-TTAGGGCACTGCGGAATCTG-3′
MMP-2	5′-ACACTGGGACCTGTCACTCC-3′	5′-TGTCACTGTCCGCCAAATAA-3′
MMP-9	5′-GCCTGTGTACACCCACATT-3′	5′-TACAGGGCCCCTTCCTTACT-3

HIF-1α, hypoxia inducible factor-1α; TGF-β1, transforming growth factor-β1; TGFβR1, TGF-β receptor type I; TGFβR2, TGF-β receptor type 2; CTGF, connective tissue growth factor; Col-1a, collagen type I-α; Col-IVa, collagen type IV-α; MMP, matrix metalloproteinase.

#### Statistical analysis.

Data are expressed as means ± SE. Multiple comparisons were performed using two-way ANOVA followed by a Student’s *t* test with post hoc Bonferroni’s correction. All statistical analysis was done using JMP software (JMP pro, version 14, SAS, Cary, NC).

## RESULTS

### 

#### Study animals.

Forty mice underwent nephrectomy, of which four mice died after nephrectomy and two mice died during contrast administration. After 28 days of nephrectomy, mice were randomized to CI or saline groups (*n* = 16 mice/group; Fig. 1). As a routine practice, body weight was recorded on the day of contrast administration (*day 0* of contrast administration). There was no significant difference in body weights between the two groups at any time point (data not shown).

#### Effect of contrast administration on kidney function.

**Fig. 2.**
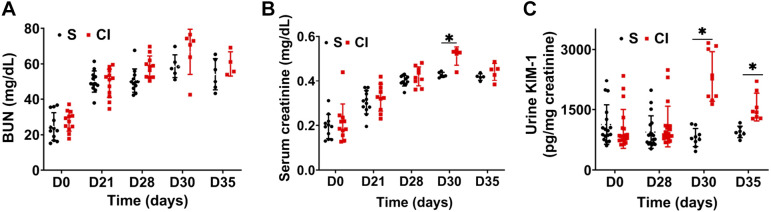
Scatterplot of kidney function in mice after intra-arterial contrast (CI group) or saline (S group) administration. *A*: average blood urea nitrogen (BUN). *B* and *C*: average serum creatinine (*B*) and average urinary kidney injury molecule-1 (KIM-1; *C*) levels in mice measured at *day 0* (D0; baseline), *day 21* (D21; 21 days after nephrectomy), *day 28* (D28; 28 days after nephrectomy), *day 30* (D30; 2 days after contrast or saline administration), and *day 35* (D35; 7 days after contrast or saline administration). Each data point represents the mean ± SD. Two-way ANOVA with a Student *t* test was performed. A significant difference between the saline and CI groups at each time point is indicated by *P < 0.05.

To evaluate the effect of intra-arterial contrast administration on kidney function, SCr and BUN were assessed at 2 days [postnephrectomy *day 30* (D30)] and 7 days [postnephrectomy *day 35* (D35)] after contrast or saline administration. There was no significant difference in average BUN at 2 days (D30) or 7 days (D35) after contrast administration compared with the saline group (Fig. 2*A*). Average SCr was significantly increased at 2 days (D30) in the CI group compared with the saline group (CI group: 0.52 ± 0.0004 mg/dL and saline group: 0.43 ± 0.003 mg/dL, average increase: 22%, *P* = 0.007). There was no difference at *day 7* (D35) between the two groups (CI group: 0.46 ± 0.0005 mg/dL and saline group: 0.42 ± 0.004 mg/dL, average increase: 11%, *P* = 0.25; Fig. 2*B*). There was a significant increase in average urinary KIM-1 levels at *day 2* (D30) in the CI group compared with saline group (CI group: 1563 ± 122 pg/mg creatinine and saline group: 809 ± 75 pg/mg creatinine, average increase: 267%, *P* < 0.0001; Fig. 2*C*). However, at *day 7* (D35), there was a decrease in average KIM-1 levels in the CI group compared with the CI group at *day 2* (D30), but it remained higher compared with the saline group at *day 7* (D35) (CI group: 2,166 ± 243 pg/mg creatinine and saline group: 1,013 ± 61 pg/mg creatinine, average increase: 154%, *P* = 0.05).

#### Contrast administration differentially activates SMAD3 in the renal cortex and medulla.

**Fig. 3.**
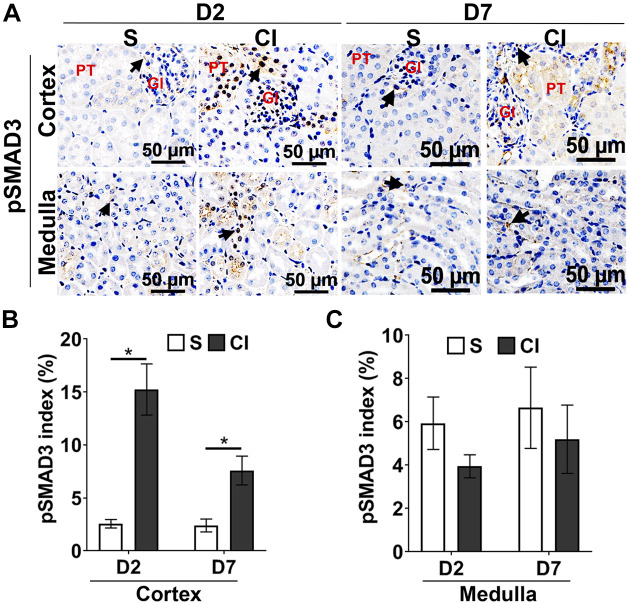
Contrast exposure increases phosphorylated (p)SMAD3 in the kidney. *A*: representative images showing immunostaining for pSMAD3 in the kidney cortex or medulla sections at *day 2* (D2) or *day 7* (D7) after contrast (CI group) or saline (S group) administration. All images were captured at ×200 magnification. Gl denotes the location of the glomerulus; PT denotes the location of the proximal tubule. Arrowheads show cells positive for pSMAD3 stain. *B* and *C*: pSMAD3 index for the cortex (*B*) and medulla (*C*). Each bar represents the mean ± SE of ≥4 animals/group. Two-way ANOVA with a Student *t* test with Bonferroni’s correction was performed. A significant difference between the saline and CI groups at each time point is indicated by *P < 0.05.

We assessed SMAD3 activation in kidney sections by immunostaining for pSMAD3 (Fig. 3*A*). A significant increase in pSMAD3 staining was found in the cortical region of the kidney from the CI group compared with the saline group of mice at 2 days (CI group: 15.24 ± 2.42 and saline group: 2.56 ± 0.46, average increase: 595%, *P* < 0.0001; Fig. 3*B*) and at 7 days (CI group: 7.58 ± 1.35 and saline group: 2.4 ± 0.66, average increase: 320%, *P* = 0.031; Fig. 3*B*) after contrast administration. Most of the nuclei staining positive for pSMAD3 were present in the tubular region. However, there was no difference in pSMAD3 staining between the CI and saline groups at *days 2* and *7* (Fig. 3*C*) in the medulla.

#### Contrast administration upregulates hypoxia-inducible factor-1α in the kidney.

**Fig. 4.**
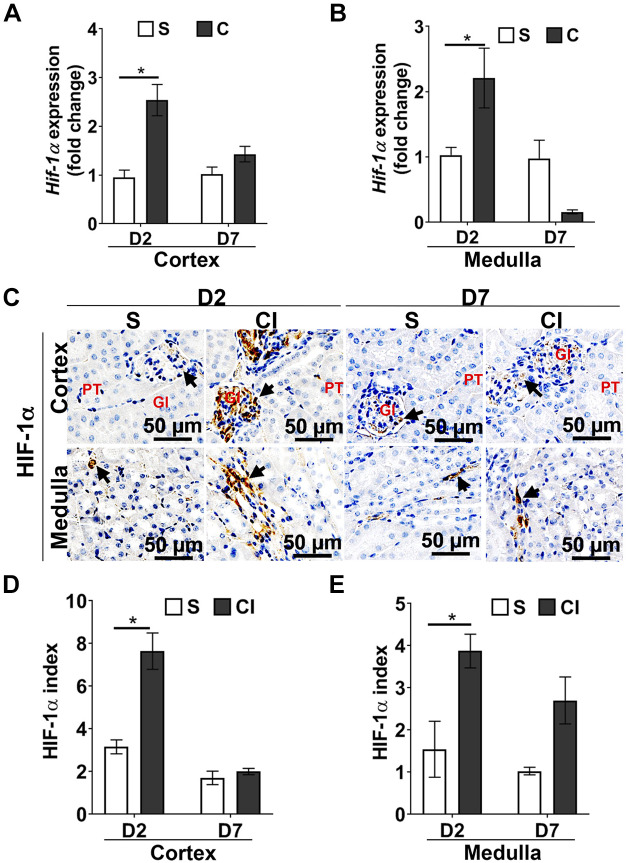
Contrast exposure increases hypoxia-inducible factor (HIF)-1α levels in the kidney. Using quantitative RT-PCR, mRNA expression of HIF-1α was determined in the renal cortex (*A*) and medulla (*B*). There was a significant increase in the average gene expression of HIF-1α in the cortex and medulla at *day 2* (*P* < 0.05). *C*: representative immunostaining images showing HIF-1α staining in the kidney cortex or medulla at *day 2* (D2) or *day 7* (D7) after contrast (CI group) or saline (S group) administration. All images were captured at ×200 magnification. Gl represents the glomerulus; PT represents the proximal tubules. Arrowheads indicate brown cells positive for HIF-1α. *D* and *E*: the HIF-1α-positive index for the cortex (*D*) and medulla (*E*). Each bar represents the mean ± SE of ≥5 animals/group. Two-way ANOVA with a Student’s *t* test with Bonferroni’s correction was performed. A significant difference between the saline and CI groups at each time point is indicated by *P < 0.05.

It is hypothesized that contrast administration causes vasoconstriction leading to hypoxic injury and a subsequent increase in hypoxia-inducible factor (HIF)-1α signaling in the kidney (8, 9). To determine whether intra-arterial contrast can cause an increase in the gene expression of HIF-1α, we performed quantitative real-time PCR. HIF-1α expression in the kidney cortex was significantly higher in the CI group compared with the saline group at *day 2* (CI group: 2.54 ± 0.32 and saline group: 1.00 ± 0.15, average increase: 254%, *P* < 0.0001; Fig. 4*A*). In the medulla, there was a significant increase in HIF-1α expression at *day 2* in the CI group compared with the saline group (CI group: 2.21 ± 0.45 and saline group: 1.02 ± 0.12, average increase: 214%, *P* = 0.005; Fig. 4*B*). We next performed immunostaining for HIF-1α in the kidney at *days 2* and *7* after saline and contrast administration (Fig. 4*C*). We observed that there was a significant increase in HIF-1α staining in the kidney cortex at 2 days after contrast administration compared with the saline group (CI group: 7.64 ± 0.94 and saline group: 3.16 ± 0.36, average increase: 241%, *P* < 0.0001; Fig. 4*D*). However, there was no difference in HIF-1α staining in the cortex at 7 days after contrast administration compared with the saline group. Most of the cortical HIF-1α staining was found in the glomerular and proximal tubular regions of the nephron, which may be attributed to contrast-mediated ischemia (27). As the contrast is cleared from the kidney by *day 7*, HIF-1α levels decreased at *day 7*. Similarly, there was a significant increase in average HIF-1α staining in the medulla of mouse kidneys of the CI group compared with the saline group at *day 2* (CI group: 3.87 ± 0.39 and saline group: 1.87 ± 0.94, average increase: 206%, *P* = 0.02; Fig. 4*D*), and the magnitude was reduced at *day 7* (CI group: 2.70 ± 0.55 and saline group: 1.61 ± 0.96, average increase: 262%, *P* = 0.06; Fig. 4*E*).

#### Contrast administration upregulates profibrotic genes in the kidney.

**Fig. 5.**
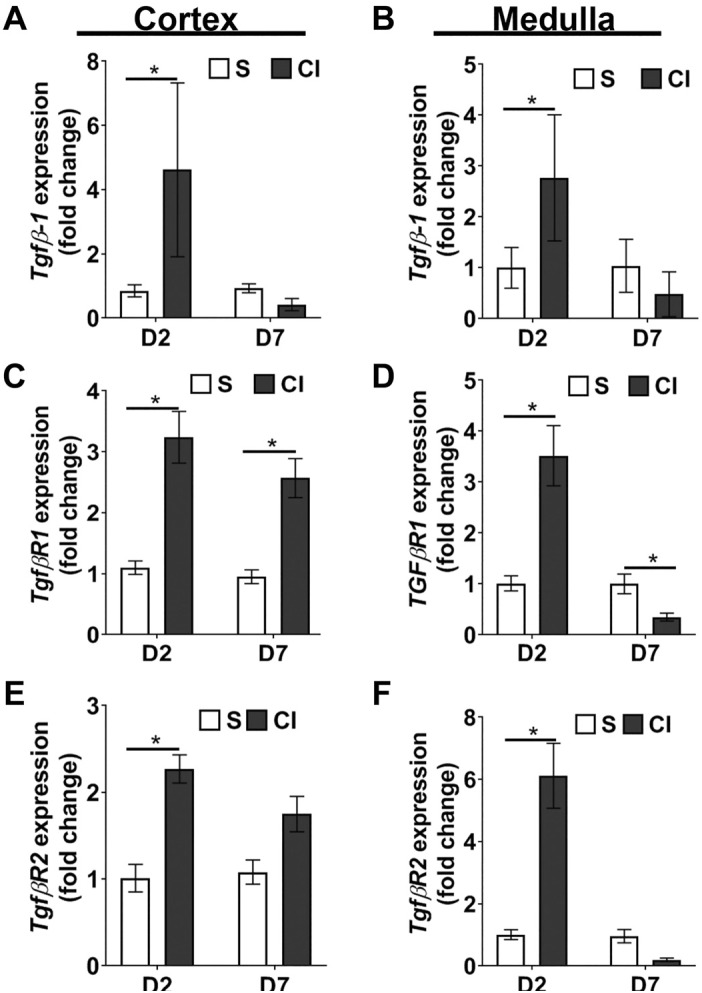
Contrast exposure increases gene expression of transforming growth factor (TGF)-β1, TGF-β receptor type I (TGFβR1), and TGF-β receptor type II (TGFβR2). Quantitative RT-PCR for mRNA expression was performed in the kidney cortex (*A*, *C*, and *E*) and medulla (*B*, *D*, and *F*). *A−F*: Tgf-β1 (*A* and *B*), TGFβR1 (*C* and *D*), and TGFβR2 (*E* and *F*) at *day 2* (D2) or *day 7* (D7) after contrast (CI group) or saline (S group) administration. Each bar represents the mean ± SE of ≥5 animals/group. Two-way ANOVA with a Student’s *t* test with Bonferroni’s correction was performed. A significant difference between the saline and CI groups at each time point is indicated by *P < 0.05.

Studies have shown that TGF-β1 contributes to the upregulation of fibrotic genes (5, 6, 39). Quantitative real-time PCR was used to determine the expression of *Tgf-β1* and other profibrotic genes in the cortex and medulla at 2 or 7 days after contrast or saline administration (Fig. 5). Average *Tgf-β1* expression was significantly increased in the renal cortex of the CI group compared with the saline group at *day 2* (CI group: 4.62 ± 1.02 and saline group: 1.00 ± 0.07, average increase: 462%, *P* < 0.0001; Fig. 5*A*), but it decreased by *day 7*. Medullary *Tgf-β1* expression was significantly increased in the CI group compared with the saline group at *day 2* (CI group: 2.77 ± 0.55 and saline group: 1.00 ± 0.15, average increase: 277%, *P* < 0.0001; Fig. 5*B*), and it decreased at 7 days after contrast administration.

We next determined the expression of TGF-β1 receptors because TGF-β binds to TGF-β1 receptor type II (TgfβR2) and triggers autophosphorylation of TGF-β1 receptor type I (TgfβR1) (28). *TgfβR1* expression in the cortex was significantly higher in the CI group compared with the saline group at *day 2* (CI group: 3.24 ± 0.39 and saline group: 1.00 ± 0.11; average increase: 324%, *P* < 0.0001; Fig. 5*C*) and *day 7* (CI group: 2.57 ± 0.29 and saline group: 1.00 ± 0.09, average increase: 257%, *P* = 0001; Fig. 5*C*). Average *TgfβR1* expression in the medulla was significantly higher in the CI group compared with the saline group at *day 2* (CI group: 3.52 ± 0.59 and saline group: 1.01 ± 0.16, average increase: 347.28%, *P* = 0.0038; Fig. 5*D*). However, average *TgfβR1* expression was significantly decreased in the medulla of the CI group compared with the saline group at 7 days (CI group: 0.35 ± 0.08 and saline group: 1.00 ± 0.19, average decrease: 65.3%, *P* = 0.01; Fig. 5*D*). We next assessed TGFβR1 protein levels by performing immunostaining. In the cortex, the average TGFβR1 index was significantly higher in the CI groups compared with the saline group at *day 2* (CI group: 50.91 ± 3.053 and saline group: 36.14 ± 2.98, average increase: 140.88%, *P* = 0.0085; Supplemental Fig. S2, *A* and *B*, available online at https://doi.org/10.6084/m9.figshare.11927127.v1; all Supplemental Data is provided at this site) and at *day 7* (CI group: 64.77 ± 2.76 and saline group: 33.01 ± 7.78, average increase: 196.22%, *P* = 0.01; Supplemental Fig. S1, *A* and *B*). In the medulla, the average TGFβR1 index was significantly higher in the CI group compared with the saline group at *day 2* (CI group: 52.69 ± 3.60 and saline group: 36.40 ± 5.49, average increase: 144.75%, *P* = 0.038; Supplemental Fig. S1, *A* and *C*), with no significant difference at *day 7*. Average *TgfβR2* was significantly increased in the cortex of the CI group compared with the saline group at 2 days (CI group: 2.27 ± 0.16 and saline group: 1.01 ± 0.13, average increase: 224%, *P* = 0.0003; Fig. 5*E*) and in the medulla (CI group: 6.11 ± 1.04 and saline group: 1.00 ± 0.15, average increase: 610%, *P* < 0.0001; Fig. 5*F*).

**Fig. 6.**
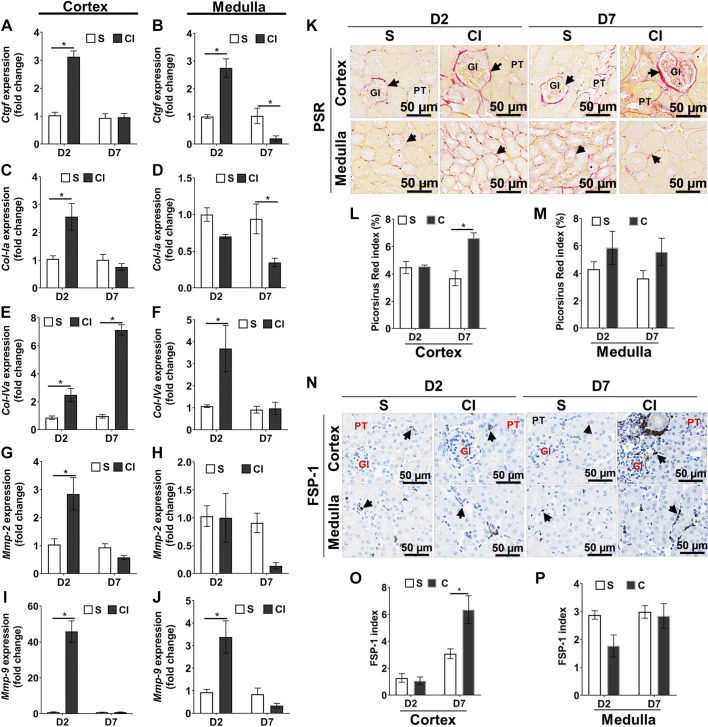
Contrast exposure increases expression and induces fibrosis in the kidney. Quantitative RT-PCR for mRNA analysis of profibrotic genes was performed for the kidney cortex (*A*, *C*, *E*, *G*, and *I*) and medulla (*B*, *D*, *F*, *H*, and *J*). *A−J*: connective tissue growth factor (*Ctgf*; *A* and *B*); collagen type I-α (*Col-Ia*; *C* and *D*), collagen type IV-α (*Col-IVa*; *E* and *F*), matrix metalloproteinase-2 (*Mmp-2*; *G* and *H*), and matrix metalloproteinase-9 (*Mmp-9*; *I* and *J*) at *day 2* (D2) or 7 (D7) after contrast (CI group) or saline (S group) administration. *K* and *N*: representative immunostaining images showing tissue fibrosis using picrosirus red staining (PSR; *K*) and fibroblast-specific protein (FSP)-1 staining (*N*) at *day 2* or *day 7* after contrast or saline administration. All images were captured at ×200 magnification. Arrowheads indicate pink stain positive for PSR, representing collagen deposition (*K*), and FSP-1-positive cells have brown staining, representing the cytoplasm (*N*). Gl represents the glomerulus; PT represents the proximal tubule. *L* and *M*: the PSR index for the cortex (*L*) and medulla (*M*). *O* and *P*: the FSP-1 index for the cortex (*O*) and medulla (*P*). Each bar represents the mean ± SE of ≥5 animals/group. Two-way ANOVA with a Student’s *t* test with Bonferroni’s correction was performed. A significant difference between the saline and CI groups at each time point is indicated by *P < 0.05.

*Tgf-β1* and tissue hypoxia can upregulate connective tissue growth factor (*Ctgf*) expression, and, therefore, we determined the gene expression of *Ctgf*. Average *Ctgf* expression was significantly increased at *day 2* in the cortex (CI group: 3.13 ± 0.206 and saline group: 1.00 ± 0.111, average increase: 313%, *P* < 0.0001; Fig. 6*A*) and in the medulla (CI group: 2.74 ± 0.33 and saline group: 1.00 ± 0.07, average increase: 275%, *P* = 0.004; Fig. 6*B*) of the kidney from the CI group compared with the saline group. Average *Ctgf* expression was significantly decreased in the medulla of the kidney from the CI group compared with the saline group at 7 days (*P* < 0.05).

Increased profibrotic genes can cause matrix deposition and tissue fibrosis (18, 23). By *day 2*, collagen type I-α (*Col-Ia*) was significantly increased in the renal cortex of the CI group compared with the saline group (CI group: 2.56 ± 0.48 and saline group: 1.02 ± 0.11, average increase: 255%, *P* = 0.001; Fig. 6*C*) but not in the medulla. However, there was a significant reduction in *Col-Ia* expression in the medullary portion of the kidney from the CI group compared with the saline group at *day 7* (CI group: 0.36 ± 0.05 and saline group: 1.02 ± 0.16, average decrease: 63%, *P* = 0.0005; Fig. 6*D*). In the cortical region of the kidney, collagen type IV-α (*Col-IVa*) expression was significantly increased in the CI group compared with the saline group at *day 2* (CI group: 2.48 ± 0.37 and saline group: 0.88 ± 0.374, average increase: 288%, *P* = 0.034; Fig. 6*E*) and *day 7* (CI group: 7.13 ± 0.35 and saline group: 0.98 ± 0.13, average increase: 713%, *P* < 0.0001; Fig. 6*E*) after contrast or saline administration. In the medullary region, average gene expression of *Col-IVa* was significantly increased in the CI group compared with the saline group at *day 2* only (CI group: 3.70 ± 1.05 and saline group: 1.08 ± 0.06, average increase: 346%, *P* = 0.028; Fig. 6*F*).

We next analyzed the gene expression of matrix metalloproteinases (MMPs). Gene expression of *Mmp-2* was significantly increased in the kidney cortex of the CI group compared with the saline group at *day 2* (CI group: 2.85 ± 0.57 and saline group: 1.04 ± 0.21, average increase: 272%, *P* < 0.0001; Fig. 6*G*). However, contrast administration had no significant impact on *Mmp-2* expression in the medulla at *day 2* or *day 7* of contrast administration (Fig. 6*H*). Average gene expression of *Mmp-9* was significantly increased in the kidney cortex (CI group: 45.81 ± 5.98 and saline group: 1.00 ± 0.26, average increase: 4531%, *P* < 0.0001; Fig. 6*I*) and in the medulla (CI group: 3.39 ± 0.79 and saline group: 1.00 ± 0.13, average increase: 339%, *P* = 0.007; Fig. 6*J*) of the CI group compared with the saline group at *day 2*.

PSR staining and immunostaining for fibroblast specific protein (FSP)-1 in kidney sections was performed to assess tissue fibrosis (Fig. 6, *K* and *N*). In the cortical portion of the kidney, by *day 7*, there was a significant increase in PSR staining in the CI group compared with the saline group (CI group: 6.61 ± 0.44 and saline group: 3.37 ± 0.55, average increase: 196%, *P* = 0.001; Fig. 6*L*). PSR was positive around the glomerular and interstitial areas in proximal tubules (Fig. 6*C*). There was a significant increase in the FSP-1 index in the cortical region of kidney sections of the CI group compared with the saline group at *day 7* (CI group: 6.35 ± 0.58 and saline group: 3.08 ± 0.20, average increase: 206%, *P* = 0.003; Fig. 6*O*). FSP-1-positive cells were found near the glomerulus and around the proximal tubular area of the cortex (Fig. 6*N*).

#### Effect of contrast administration on cell proliferation and apoptosis in the kidney.

**Fig. 7.**
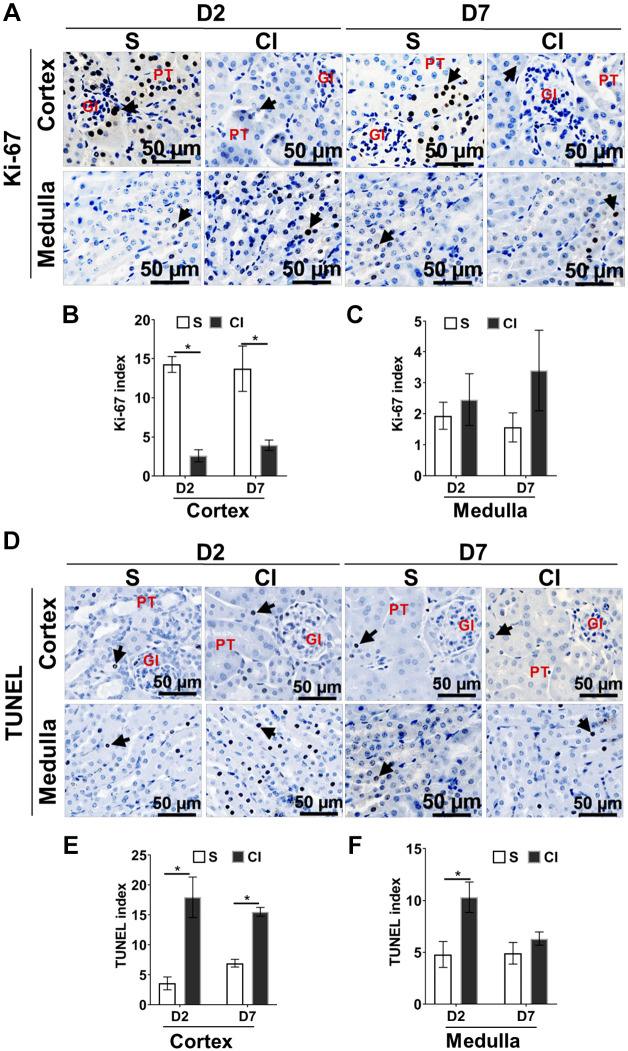
Contrast administration inhibits proliferation and induces apoptosis in the kidney. *A* and *D*: cell proliferation (*A*) and apoptosis (*D*) was determined by immunostaining for Ki-67 and TUNEL staining, respectively. Representative images of Ki-67 (*A*) and TUNEL (*D*) staining in the kidney cortex or medulla at *day 2* (D2) or *day 7* (D7) after contrast (CI group) or saline (S group) administration are shown. All images were captured at ×200 magnification. Ki-67-positive cells have brown-stained nuclei. TUNEL-positive cells have brown-stained nuclei. Gl represents the glomerulus; PT represents the proximal tubule. Arrowheads indicate brown stain. *B* and *C*: the Ki-67-positive index for the cortex (*B*) and in the medulla (*C*). *E* and *F*: the TUNEL index for the cortex (*E*) and medulla (*F*). Each bar represents the mean ± SE of ≥5 animals/group. Two-way ANOVA with Student’s *t* test with Bonferroni’s correction was performed. A significant difference between the saline and CI groups at each time point is indicated by *P < 0.05.

We evaluated the effect of contrast administration on cell proliferation (Ki-67) and apoptosis (TUNEL). We observed that there was a significant decrease in the average Ki-67 index in the cortical region of the kidney from the CI group compared with the saline group at *day 2* (CI group: 2.60 ± 0.77 and saline group: 14.26 ± 1.13, average decrease: 548%, *P* < 0.0001; Fig. 7, *A* and *B*) and *day 7* (CI group: 3.94 ± 0.66 and saline group: 13.72 ± 3.70, average decrease: 442%, *P* < 0.0001; Figs 7, *A* and *B*). Ki-67-positive cells were located near the proximal tubular epithelial cells in the saline group (Fig. 7*A*).

We next assessed cell apoptosis by performing TUNEL staining (Fig. 7*D*). There was a significant increase in the TUNEL index in the cortical region of the kidney of the CI group compared with the saline group at *day 2* (CI group: 17.96 ± 3.78 and saline group: 3.60 ± 1.19, average increase: 499%, *P* < 0.0001; Fig. 7*E*) and at *day 7* (CI group: 15.53 ± 0.95 and saline group: 6.97 ± 0.64, average increase: 222%, *P* = 0.026; Fig. 7*E*). TUNEL-positive cells were mostly localized in the proximal tubule and glomerular region of the nephron. There was a significant increase in average TUNEL staining in the medullary region at *day 2* in the CI group compared with the saline group (CI group: 10.32 ± 1.47 and saline group: 4.80 ± 1.61, average increase: 222%, *P* = 0.026; Fig. 7*F*).

#### Contrast administration downregulates E-cadherin staining in the kidneys.

To determine whether the increase in TUNEL staining was due to a decrease in epithelial cells, we stained kidney sections from the cortex and medulla with E-cadherin. In the cortex, the average amount of cells staining positive for E-cadherin was significantly decreased in the CI group compared with the saline group at 2 days (CI group: 3.39 ± 0.69 and saline group: 8.46 ± 1.56, average decrease: 60%, *P* = 0.03) and at 7 days (CI group: 2.17 ± 0.43 and saline group: 6.33 ± 1.03, average decrease: 66%, *P* = 0.0058; Supplemental Fig. S2, *A* and *B*). However, in the medulla, there was no significant difference in E-cadherin staining at 2 or 7 days after contrast administration compared with the saline group (Supplemental Fig. S2, *A* and *C*).

## DISCUSSION

In the present study, we created a murine model of CI-AKI by intra-arterial contrast agent administration in mice with established CKD. There was a significant increase in SCr by 22% accompanied with an increase in urinary KIM-1 levels at 48 h after contrast administration. Histological examination indicated that there was an increase in pSMAD3 levels in the cortex of mice at 48 h after contrast administration, and it remained increased at 7 days compared with saline controls. There was also an increase in HIF-1α, fibrotic genes, and collagen deposition, accompanied by a decrease in proliferation and increase in apoptosis. These results are consistent with a previous study (14) in the CKD mouse model with intravenous contrast administration, suggesting that contrast administration impairs kidney function and increases tissue fibrosis despite the route of contrast administration.

There are conflicting studies on whether intra-arterial contrast administration is more nephrotoxic compared with the intravenous route of contrast administration. Some studies have demonstrated that intra-arterial administration is worse possibly because of other factors, such as atheroemboli (26, 31) Other clinical studies have demonstrated that CI-AKI is independent of the route of contrast administration (12, 21). Multiple hypotheses have been suggested for the etiology of the toxicity of contrast following intra-arterial or intravenous administration (4). However, the mechanisms responsible for CI-AKI after intra-arterial contrast administration remain poorly understood because of the lack of experimental animal models. One theory is that contrast causes a transient vasoconstriction and ischemic injury, resulting in fibrosis and apoptosis (4). However, CI-AKI in patients with other risk factors, such as CKD, can lead to renal interstitial fibrosis that results in a gradual loss of renal function (30).

In our 5/6 nephrectomy CKD model, there was a significant increase in average SCr and BUN levels at 4 wk after nephrectomy compared with baseline. However, there was no significant change in average urinary KIM-1 levels after CKD at the same time points, suggesting that there was no tubular damage (15, 32). We observed an increase in average SCr with an increase in urinary KIM-1 levels at 2 days after contrast administration. The rise in KIM-1 levels suggests that there is evidence of tubular damage in the remnant kidney.

In the present study, we observed an upregulation of *Tgf-β1* and its receptors *TgfβR1* and *TgfβR2* as well as activation of SMAD3 signaling following intra-arterial contrast administration. These results are consistent with the studies in a CKD mouse model with intravenous contrast administration, and blockade of TGF-β1/SMAD3 signaling abrogates contrast-mediated expression of fibrotic genes in HK2 cells (a proximal tubular cell line derived from the human kidney) (14). Moreover, increased pSMAD3 staining was observed predominantly in the nuclei of proximal tubular cells of the kidney cortex at 2 and 7 days after contrast administration. These results further support the notion that proximal tubular cells of the cortex are affected early by CI-AKI (24).

Tissue hypoxia and vasoconstriction can also be attributed to CI-AKI in vivo (16). Therefore, HIF-1α staining was performed to assess the extent of tissue hypoxia in kidney sections. HIF-1α staining was increased at *day 2* in both the cortex and medulla, suggesting that hypoxic injury could be an early event followed by TGF-β1/SMAD signaling activation in CI-AKI. Studies have shown that HIF-1α, along with increased TGF-β1 signaling, contributes to tissue fibrosis and upregulation of profibrotic genes (5, 6, 39). Intra-arterial contrast administration led to a significant increase in *Ctgf* gene expression in the cortex and medulla after 2 days of contrast administration. Tgf-β1 increases *Ctgf* expression, which attenuates cell proliferation and exacerbates tissue fibrosis via upregulation of extracellular matrix proteins (33). In the present study, there was a decrease in cell proliferation with an increase in cell death in the kidney cortex 2 and 7 days after contrast administration compared with saline administration. However, there is an increase in cell death in the medulla, with no effect on cell proliferation at 2 days after contrast administration, which could be due to an increase in tissue hypoxia (19).

Moreover, an important observation in the present study is that most of the profibrotic genes were significantly increased at 2 days after contrast administration and were neutral or decreased at *day 7* in both the cortex and medulla except for *Col-IVa*, which is increased in renal fibrosis (3). Although fibrosis is a chronic process, an increase in profibrotic genes may be a primary response to the initial stimuli, which may decrease by 7 days after contrast administration. However, there was a significant increase in the gene expression of *Col-IVa* and *Ctgf* in the medulla at 2 days after contrast administration, despite no change in pSMAD3. This likely represents a SMAD3-independent mechanism. Studies have shown that SMAD3 knockdown could not abrogate TGF-β1-mediated *Col-IVa* expression in glomerular mesangial cells (38a, 40), suggesting that SMAD3-independent pathways of tissue fibrosis exist.

Although the induction of extracellular matrix proteins was observed in both the cortex and medulla, the effect was more pronounced in the cortical region with intra-arterial contrast administration. Such segmental effects could be due to an initial increase in blood flow to the cortex with contrast infusion (17), which may cause proximal tubular cells to be exposed to concentrated levels of contrast agent and subsequent cytotoxicity.

In the present study, *Mmp-2* upregulation was restricted to the kidney cortex, with *Mmp-9* being upregulated in both the cortex and medulla at 2 days after intra-arterial contrast agent administration. Intriguingly, *Mmp-9* expression was much higher (45-fold) compared with *Mmp-2* expression (3-fold) in the cortex. MMP-9 is involved in the degradation of collagen type IV, which leads to the disruption of the basement membrane and epithelial-to-mesenchymal transition of tubular epithelial cells (29, 38, 41). MMP-9 has also reported to be involved in the activation of TGF-β and endothelin-1 (35). Together, the higher expression of *Mmp-9* in the cortex may contribute to aggressive TGF-β1/SMAD3 signaling, possibly due to the vasoconstriction secondary to the contrast administration that could lead to tissue fibrosis.

Moreover, a reduction of cell proliferation and increase in apoptosis could be another plausible explanation for the reduction of profibrotic genes at 7 days after contrast administration. Finally, there was a significant reduction of E-cadherin, an epithelial marker, in the CI group compared with the saline group in the cortex at 2 and 7 days.

In summary, we created a murine model of intra-arterial contrast administration causing CI-AKI to simulate the clinical scenario of patients undergoing intra-arterial contrast administration with impaired renal function. In this model, we demonstrated that contrast administration activates the TGF-β1/pSMAD3 pathway, along with increased tissue hypoxia and fibrosis in the mouse kidney. Moreover, cortical and medullary segments of the kidney had differential responses in terms of activation and expression of TGF-β/pSMAD3 pathway and intermediates that led to an increase in fibrosis and apoptosis with a decrease in proliferation. Although further studies are required to study CI-AKI, the results from this study further support the notion that similar signaling cues are involved in CI-AKI, irrespective of the intravascular route of contrast administration.

## GRANTS

This work was funded by National Institutes of Health Grants HL-098967 and DK-107870 (to S. Misra).

## DISCLAIMERS

The National Institutes of Health had no role in the study design, data collection and analysis, decision to publish, or preparation of the manuscript.

## DISCLOSURES

No conflicts of interest, financial or otherwise, are declared by the authors.

## AUTHOR CONTRIBUTIONS

S.M. conceived and designed research; A.S., S.K., C.C., and M.L.S. performed experiments; A.S., S.K., C.C., M.L.S., and S.M. analyzed data; A.S., S.K., C.C., and S.M. interpreted results of experiments; A.S., S.K., C.C., and S.M. prepared figures; A.S., S.K., C.C., and S.M. drafted manuscript; S.K., C.C., and S.M. edited and revised manuscript; S.M. approved final version of manuscript.
